# Supplementary material for: The interactome of intact mitochondria by cross-linking mass spectrometry provides evidence for coexisting respiratory supercomplexes
Source: Mol Cell Proteomics. 2017 Dec 8;17(2):216–32. doi: 10.1074/mcp.RA117.000470 (PMC5795388; doi:10.1074/mcp.RA117.000470)
Supplement: Supplemental Data [file supp_RA117.000470_133922_0_supp_23978_fzffwf.zip › spectra_annotation/mito_spectra_annotation/53-1-9-1-4-1.pdf]

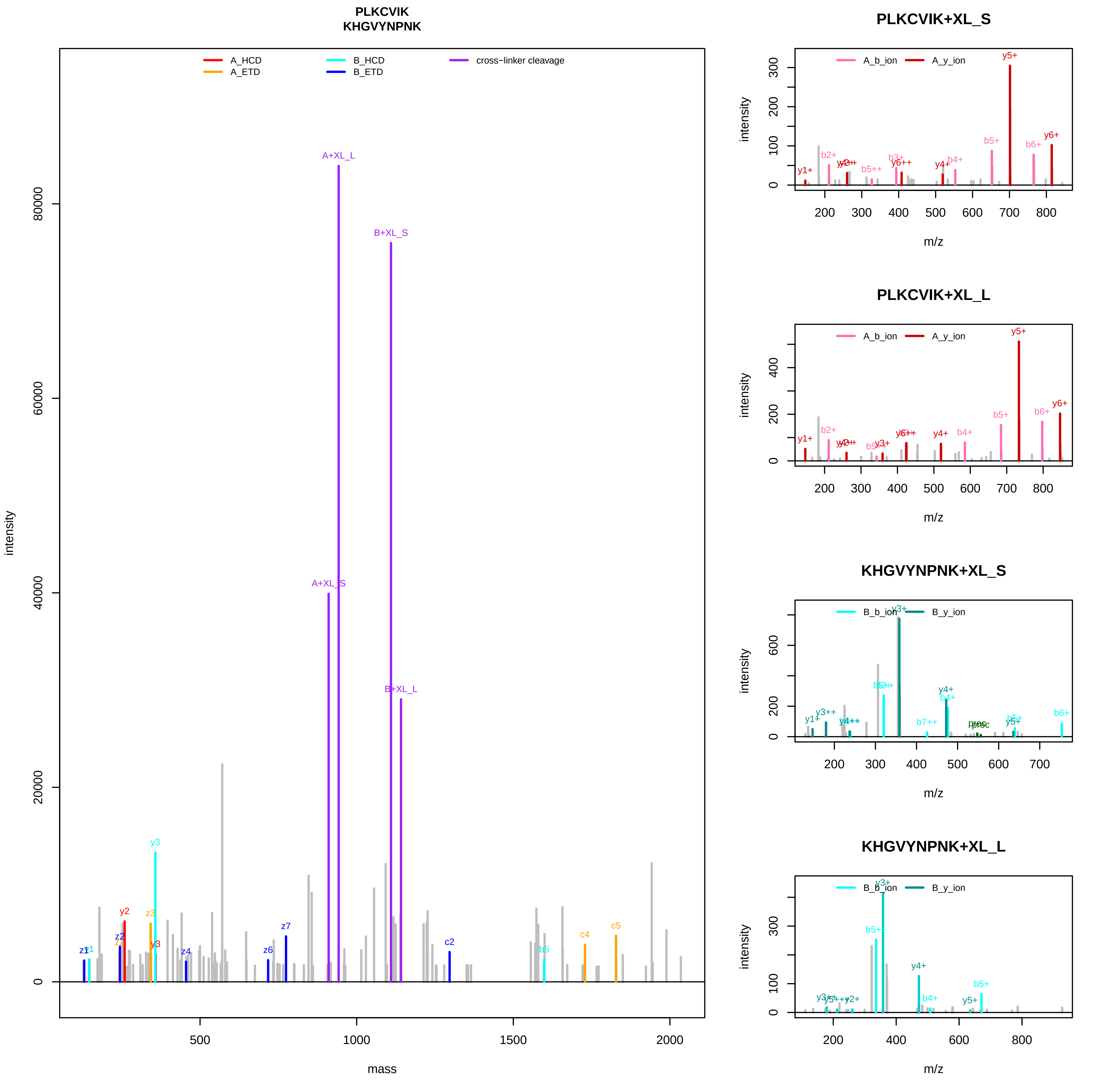
PLKCVIK
KHGVYNPNK
A_HCD
A_ETD
B_HCD
B_ETD
cross−linker cleavage
A+XL_L
B+XL_S
A+XL_S
B+XL_L
y2
y3
z1
z2
z3
z4
z6
z7
c2
c4
c5
b5
0
20000
40000
60000
80000
intensity
500
1000
1500
2000
mass
PLKCVIK+XL_S
PLKCVIK+XL_L
KHGVYNPNK+XL_S
KHGVYNPNK+XL_L
A_b_ion
A_y_ion
B_b_ion
B_y_ion
y1+
b2+
y2++
b3+
b4+
b5+
b6+
b5++
y3+
y4+
y5+
y6+
y6++
y1+
y3++
y4++
b7++
y4+
b4+
y5+
b5+
b6+
prec
y3+
b5++
y2+
y5++
intensity
m/z
0
100
200
300
400
600
700
800
